# Streamlining Quantitative Analysis of Long RNA Sequencing Reads

**DOI:** 10.3390/ijms21197259

**Published:** 2020-10-01

**Authors:** Sebastian Oeck, Alicia I. Tüns, Sebastian Hurst, Alexander Schramm

**Affiliations:** 1 Department of Medical Oncology, West German Cancer Center, University Hospital Essen, University of Duisburg-Essen, 45147 Essen, Germany; alicia.tuens@uk-essen.de; 2 Institute of Cell Biology, University of Münster, 48149 Münster, Germany; sebastian.hurst@uni-muenster.de

**Keywords:** Alignator, long reads, alignment, RNA, FASTQ, sequencing tool, index, Oxford Nanopore Technology, PacBio

## Abstract

Transcriptome analyses allow for linking RNA expression profiles to cellular pathways and phenotypes. Despite improvements in sequencing methodology, whole transcriptome analyses are still tedious, especially for methodologies producing long reads. Currently, available data analysis software often lacks cost- and time-efficient workflows. Although kit-based workflows and benchtop platforms for RNA sequencing provide software options, e.g., cloud-based tools to analyze basecalled reads, quantitative, and easy-to-use solutions for transcriptome analysis, especially for non-human data, are missing. We therefore developed a user-friendly tool, termed Alignator, for rapid analysis of long RNA reads requiring only FASTQ files and an Ensembl cDNA database reference. After successful mapping, Alignator generates quantitative information for each transcript and provides a table in which sequenced and aligned RNA are stored for further comparative analyses.

## 1. Introduction

Deep sequencing of mRNA represents a powerful tool in cancer genome research as well as in cell biology [1,2,3]. Expression patterns that are encoded in the transcriptome enable researchers to link the effects of pathway alterations, mutations, or extrinsic stimuli to molecular responses of cells [4,5,6,7]. Despite improvements in sequencing methodology, whole genome and transcriptome analyses are still cost- and time-intensive [8,9,10,11]. In our experience, one bottleneck in transcriptome analyses is a lack of off-the-shelf solutions, slowing down the process of transcriptome sequencing analyses. Since transcriptome and proteome analyses are often used to generate a hypothesis [1,2,5,12], it is likely that problems in big data analyses are often linked to restricted access to bioinformatics core groups. Thus, creating user-friendly analyses pipelines, which is desirable in all scientific disciplines, is especially helpful in the context of transcriptome analyses.

While several solutions for analyzing RNA sequencing data using short reads (ca. 50–300 bp) are available [13], longer RNA reads as obtained from the Oxford Nanopore Technology (ONT) MinION system are still more difficult to analyze quantitatively. In contrast to conventional sequencing approaches, nanopore sequencing records conductivity changes when a DNA or RNA strand passes through a biological pore [14]. These signals can be converted to DNA or RNA sequences and can be stored as FASTQ files containing long (>2 kbp) RNA reads. For ONT technology, this process is guided by MinKNOW, an operating software that drives the nanopore sequencing device, performs raw data acquisition, and stores the fluctuating electrical signals in FAST5 files. MinKNOW also allows for local base calling, a process which translates the raw signals into base sequences that are stored in FASTQ files [12], which can be immediately processed by the user allowing almost real-time sequencing. The cloud-based ONT platform EPI2ME is readily applicable to analyze human and bacterial DNA reads. However, solutions for mRNA analysis, especially for non-human data, are missing. Therefore, we developed an easy-to-use tool for rapid analysis of long RNA reads, implementing the seed-and-vote algorithm of the Subread package for R [15] and making use of our previously developed high-throughput tools [16,17]. This tool, which we termed Alignator, is principally suited to quantitatively describe, filter, and analyze any data set consisting of long RNA reads from a self-explanatory user interface.

## 2. A General Workflow for Analyzing Long RNA Reads

**Figure 1:**
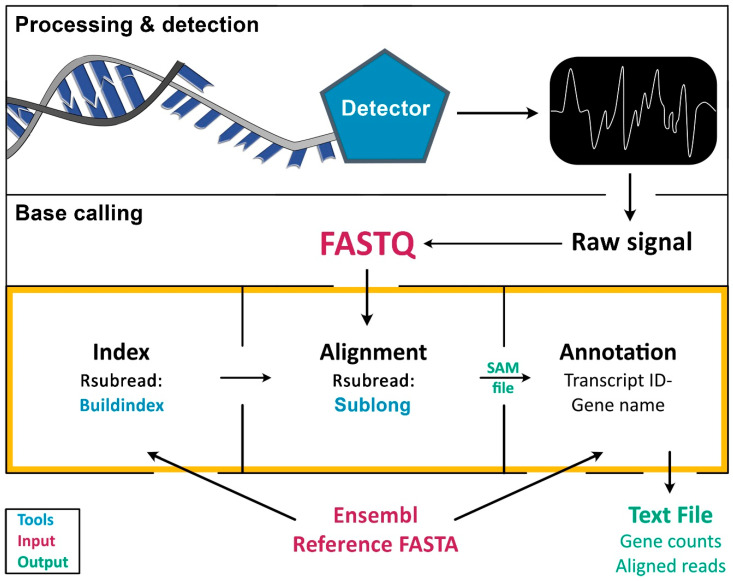
**Principle of sequencing data processing and implementation of Alignator v1.** The process of RNA sequence analysis is divided into three major steps: following RNA isolation, raw signals are obtained during RNA sequencing (Processing & detection). Raw signals are then converted into sequence data (Base calling), which can be aligned and quantified. The orange frame marks the tasks implemented in Alignator v1, including index generation, alignment, and annotation of the aligned reads. Tools that are used by Alignator v1 are marked in blue, whereas required input files are depicted in red. Successful runs will produce the three output files shown in green.

To extend existing pipelines for long sequencing reads to the RNA level, our approach was to integrate existing workflows for base calling, alignments, and indexing to enable the generation of an easily accessible data (text) file containing both gene counts and aligned RNA reads. The resulting analyses tool, Alignator, is readily available and easy-to-use for FASTQ files obtained from long RNA reads such as ONT as described below or PacBio [18] but is principally suited for analyses of any RNA sequencing experiments using long reads. In contrast to other technologies, MinION sequencing devices convert changes in ion current resulting from an RNA that passes through a pore into a raw signal (Figure 1, Processing & detection). This signal is translated and stored in FASTQ files (Figure 1, Base calling).

Technically, this workflow is applicable to any method that generates sequencing output in FASTQ files containing sequence information and quality metrics. To align long reads to a reference genome (FASTA file), the genome data obtained from a database (e.g., Ensembl) must be indexed first (Figure 1, orange frame). This is usually achieved using stand-alone scripts, such as Buildindex [15]. Subsequently, the index is used by an alignment algorithm, such as Sublong, to identify sequence homologies in the chosen species-specific index. The result of the alignment procedure is a SAM file, which basically contains the entire information of the alignment/mapping process, including read length and quality metrics. Finally, transcript IDs and gene names must be linked to SAM files using annotations from reference genomes to enable qualitative and quantitative gene expression analyses.

## 3. Workflow of Alignator v1

### 3.1. General Principle

One big advantage of our new alignment script is that it only requires the sequenced reads (FASTQ files) as input and an Ensembl cDNA database (FA file) as a reference. All the steps can be performed using established R scripts and packages, which are integrated in a sophisticated manner. For example, Alignator allows using the same Ensembl cDNA file for alignment (via Rsubread) and annotation of the generated SAM file. Here, the reference genome is compressed into an index file using Buildindex, which is part of the Rsubread Bioconductor package [15] (Figure 1, orange frame). This compression also reduces requirements for computer memory. The alignment of the reads is performed by Sublong, an implementation of the Rsubread algorithm, which is especially suited for long reads. The output of Alignator is a file including the alignment information (e.g., gene name, sequencing quality parameters) and a file including the summed-up counts of reads per gene for further quantitative analyses.

### 3.2. Indexing and Aligning of Long Reads

**Figure 2:**
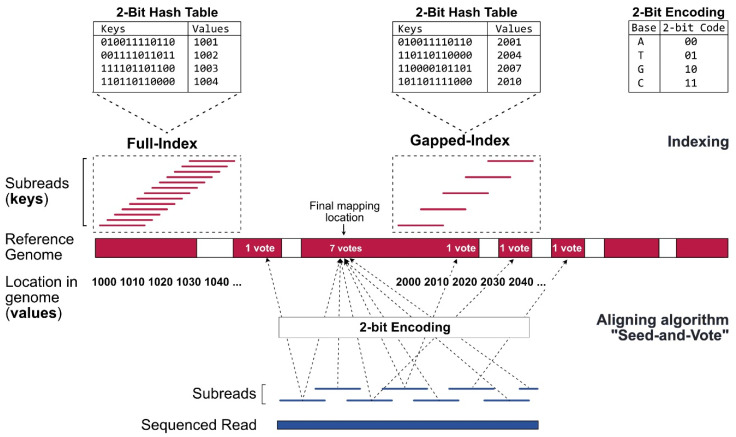
**Indexing and alignment strategy of Rsubread for long RNA reads.** The alignment strategy is based on short overlapping subreads, extracted from the sequenced reads (blue) and from the reference FASTA file (red). When determining the optimal mapping location of a long read, the seed-and-vote strategy allows the subreads (seeds) to vote for several positions (dashed arrows). The position with the highest number of votes is chosen as the final mapping location.

Since the alignment of sequencing reads against a reference genome is a rate-limiting step in the process requiring extensive computing power, genomes should be compressed into an index file (Figure 2). Alignator builds a full-index table by extracting 16 bp-mers (subreads) from every location of the reference genome using the Rsubread function Buildindex [15]. The chromosomal locations (values), as well as the 2-bit encoded (A: 00, T: 01, G: 10, C: 11) sequence information (keys) of the subreads, are recorded in a hash table. This enables faster results when searching for specific elements. Once the index is generated, it can be reused for future alignments. As a second step, the optimal mapping side of the reads is determined. The Sublong function uses the seed-and-vote strategy for aligning long reads to the reference. In brief, overlapping subreads (seeds) are extracted from every read. Afterwards, the seeds vote for optimal read locations. The location that receives the largest number of votes is picked as the final mapping location.

## 4. The User-Friendly Interface of the Alignator v1 and How to Use It

### 4.1. The Interface and Index Generation

**Figure 3:**
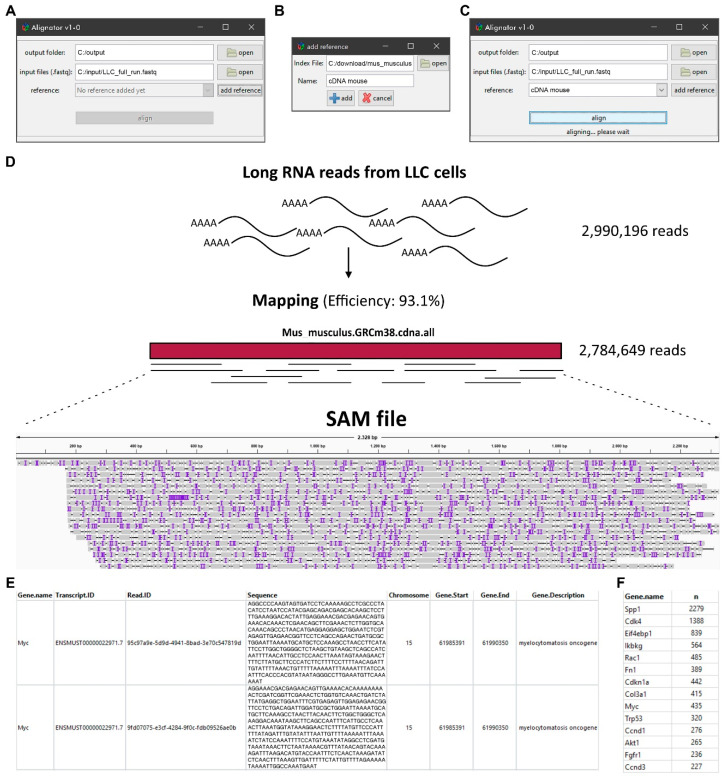
**The interface of the Alignator v1 and output files.** (**A**) Main window with options to set-up the output folder and input files as well as an option for creating the index reference via the add reference button. (**B**) The add reference window with the option to choose the index file (e.g., a local copy of the Ensembl reference genome) and to assign a customized name for the index. (**C**) The run progress in the main window after hitting the align button. Detailed information about the run can be followed in the RStudio’s console (not shown). (**D**) Exemplary long RNA read alignment of murine cell line Lewis lung carcinoma (LLC). The sequence alignment map (SAM) file graphically shows read mapping and coverage of a specific region (Myc-205). Purple lines represent inserted bases, whereas black horizontal lines show missing bases. (**E**) The AnnotatedData file contains all data—e.g., gene name, transcript ID, read sequence, chromosomal location—derived from the SAM file and the annotation file. (**F**) The compact GeneCounts file contains gene names and their corresponding counts (n) for downstream quantitative analyses.

The Alignator v1 comes with a compact and straightforward interface (Figure 3A–C) that can be run from the RStudio workspace. The Alignator R script can be found in the supplement to this manuscript. After installing the R language package (https://cran.r-project.org/) and RStudio (https://rstudio.com/), the Alignator R script (Alignator_v1-x.R) opens in RStudio and starts using the source button that opens the main window (Figure 3A). Here, the user chooses the output folder and input files. If necessary, an index reference can be created via the add reference button (Figure 3B). The add reference window gives the option to choose an Ensembl input file required to create the index. For convenience, a customized name for the index can be chosen. Furthermore, multiple indices are created for later use. After selecting a newly created or pre-existing index, output folder, and input files in FASTQ format, the run starts using the align button (Figure 3C). Progress of the run is displayed in the main window. Detailed information about the run can be followed in RStudio’s console. To evaluate the effects of parallelization on run time, we limited the number of available threats during mapping runs. This test showed that parallelization decreases run time; however, using more than four cores probably will not significantly improve processing (Supplementary Figure S1).

### 4.2. The Output Files

Successful runs generate a sequence alignment map (SAM) file and two result files stored in the predefined output folder. The SAM file saves RNA reads that were successfully aligned to a reference sequence in a text-based format. When imported into genome viewer software, the file can be used for graphical representation of the mapping. Furthermore, it contains information, such as read IDs, deletions, insertions, mismatches, and error probability for each individual base. As a representative example, the SAM file in Figure 3D visualizes long RNA reads produced from mouse Lewis lung carcinoma (LLC) cells that map to the murine MYC transcript ENSMUST00000022971.7. The AnnotatedData file (Figure 3E) contains the aligned reads of the SAM file that are linked to additional information, including gene names, chromosomal locations, and short gene descriptions. In contrast to the comprehensive AnnotatedData file, the compact GeneCounts file only contains gene names and their corresponding read counts in the respective run (Figure 3F). In our example, transcriptome analysis of the LLC cell line revealed 435 long reads derived from the murine MYC gene. As the overall read count number can also be obtained, the information in GeneCounts can be used for normalization and quantitative analyses of MYC target genes in any given sequencing run.

## 5. Conclusions

Here, we introduced an innovative and self-explanatory R-based tool, designated Alignator, for unbiased quantitative analyses of long RNA reads. Alignator enables users to directly create index files for any species for which a reference genome is publicly available. This only requires a local copy of a reference file that can be downloaded, e.g., from the Ensembl databases. Once the reference is compressed into an index file, it can immediately be used for analysis without further processing. The user interface and its output are easy to understand and intuitive. We successfully showed that the combination of the comprehensive AnnotatedData file and the compact GeneCounts file offers an easy way to obtain quantitative results on RNA abundance. This can be further used for downstream analyses of biological contexts, as shown here for LLC cells.

## 6. Materials and Methods

### 6.1. Hardware and Chemicals

The Alignator can be run on any hardware that supports R language and RStudio. A human cDNA database requires 3 to 4 Gb of RAM and about 2.3 Gb of free disk space. The MinION device and related flow cells (FLO-MIN106D; R9.4.1) were acquired from Oxford Nanopore Technologies (Oxford, UK). All other chemicals were purchased from Sigma-Aldrich (Deisenhofen, Germany).

### 6.2. RNA Sample Preparation

RNA sequencing was performed with 500 ng poly-A enriched RNA isolated with the NEBNext Poly(A) mRNA Magnetic Isolation Module (New England Biolabs, Ipswich, MA, USA) using the Direct RNA sequencing kit (SQK-RNA002, Oxford Nanopore Technologies, Oxford, UK). This procedure is feasible for high amounts of RNA (40–50 µg). For low amounts, the cDNA-PCR Sequencing kit (SQK-PCS109, Oxford Nanopore Technologies, Oxford, UK) is recommended [19]. Sample preparation was performed according to the manufacturer’s instructions.

### 6.3. Cell Culture

Murine Lewis lung carcinoma (LLC) cells were purchased from ATCC (Bethesda, MD, USA). Cells were cultivated in RPMI (Life Technologies Ltd., Paisley, UK) medium supplemented with 10% (*v*/*v*) fetal calf serum (Biowest, Nuaillé, France) and maintained in a humidified incubator (Thermo Fisher Scientific/Thermo Electron LED GmbH, Langenselbold, Germany) at 37 °C and 5% CO_2_.

### 6.4. Software

The MinKNOW 3.3.2 software is available to registered users from the Oxford Nanopore Technologies website (https://nanoporetech.com/products/minion). For analyses in the R environment, we recommend using RStudio 1.3 and R 4.0.2, or higher versions of both (R Core Team (2020) is an open-source software package. R: a language and environment for statistical computing. R Foundation for Statistical Computing, Vienna, Austria. URL https://www.R-project.org/). We used Rsubread included in the Bioconductor version 3.11 or higher [15].

## Supplementary Materials

The following figures are available online at https://www.mdpi.com/1422-0067/21/19/7259/s1. Figure S1: Benchmarking the effects of core threads used on runtime for read processing using Alignator. The two additional supplementary files contain the script of the tool as text file Alignator_v1-1.txt and as R-Script Alignator_v1-1.R.
