# Supplementary figures and images for: Streamlining Quantitative Analysis of Long RNA Sequencing Reads
Source: Int J Mol Sci. 2020 Oct 1;21(19):7259. doi: 10.3390/ijms21197259 (PMC7584020; doi:10.3390/ijms21197259)

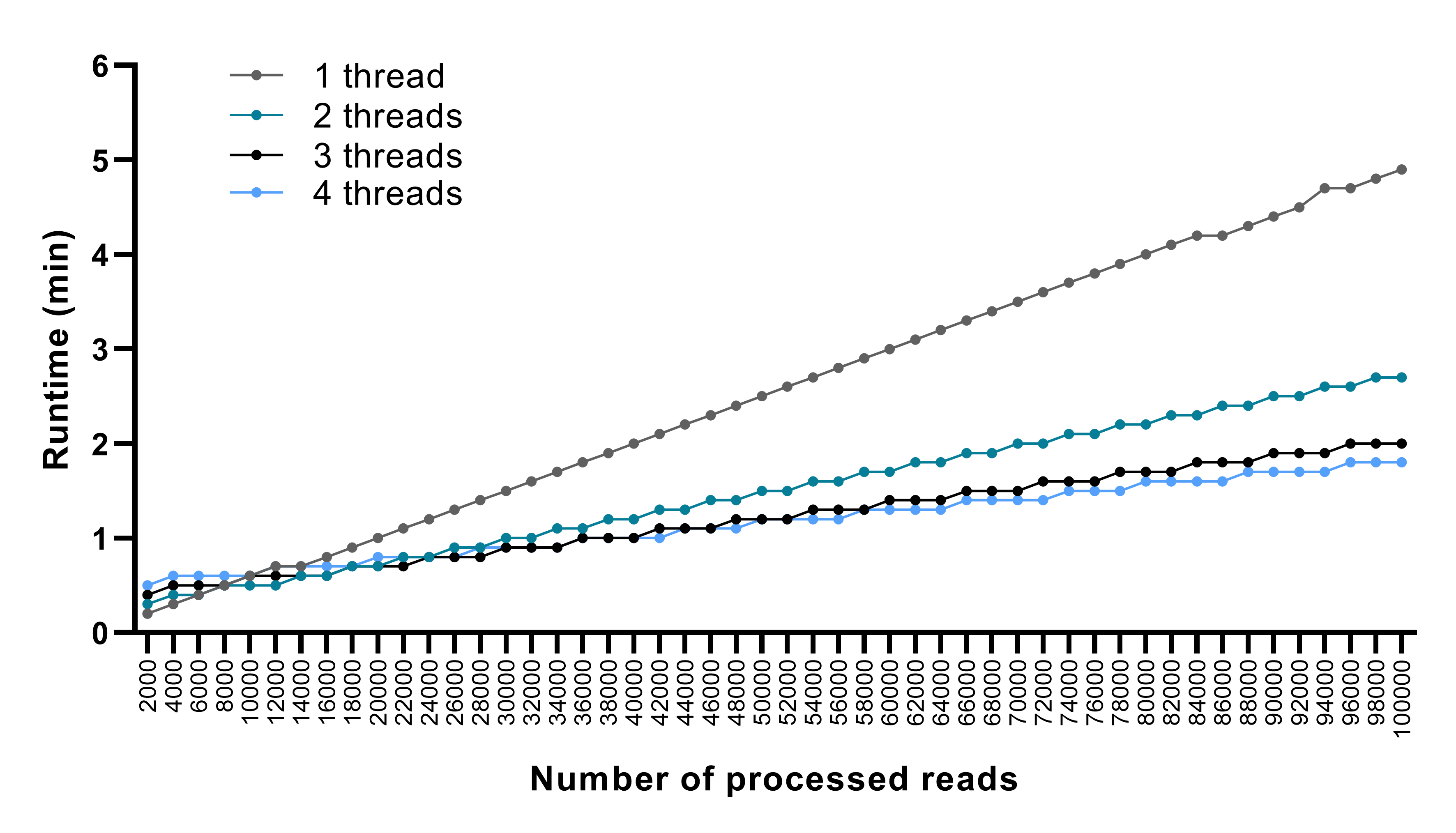

Supplement: Supplementary file 1 [file ijms-21-07259-s001.zip › ijms-938067-supplementary/Suppl Figure 1.png]
